# Leydig cell tumors of the testis: a case report

**DOI:** 10.1186/1756-0500-7-656

**Published:** 2014-09-18

**Authors:** Ancuta Augustina Gheorghisan-Galateanu

**Affiliations:** Department of Cellular and Molecular Medicine, Carol Davila University of Medicine and Pharmacy, 8 Eroii Sanitari Blvd, Bucharest, 050474 Romania; C.I.Parhon National Institute of Endocrinology, 34 Aviatorilor Blvd, Bucharest, 011853 Romania

**Keywords:** Leydig cell tumor, Overweight, Gynecomastia, Estradiol, Subdiaphragmatic micro lymphadenopathy

## Abstract

**Background:**

Leydig cell tumors are the most common non-germ cell gonadal tumors with apparent increased incidence in the last few years. They are usually benign tumors. We report a case of Leydig cell tumor of testis in a patient presenting atypical features.

**Case presentation:**

A 29-year-old Caucasian man, born with right cryptorchidism, corrected without medical treatment before the age of two years, was diagnosed with Leydig cell tumor. Two years after diagnosis was identified moderately elevated estradiol serum level, in the context of a significant overweight, hormonal changes which had maintained after unilateral orchiectomy and after the patient's return to normal weight. Four years after unilateral orchiectomy, elevated value of estradiol persisted and subdiaphragmatic micro lymphadenopathy was observed.

**Conclusions:**

Despite the favorable evolution of the patient four years after unilateral orchiectomy, long-term follow-up is necessary to exclude recurrence or metastasis to the testis. The endocrine profile and imaging investigations need to be repeated periodically. The changes in the hormonal assay and any new aspects on computed tomography scan can be used as a marker of tumor recurrence and require careful screening and the correct therapeutic decisions.

## Background

Approximately 5-6% of all testis tumors are non-germ cell tumors. Included in this group are sex cord/gonadal stromal tumors, most originating from Leydig or Sertoli cells, mixed tumors, and tumors of mesenchymal or hematopoietic origin [1].

Leydig cell tumors (LCTs) are uncommon neoplasms arising from gonadal stroma, accounting for 1-3% of all testicular tumors in adults and 4% in prepubertal children [2, 3]. In the last few years the incidence of LCTs seemed to increase well above the literature predictions (14.7% of all testicular tumors removed). One possible explanation for this phenomenon is the increasing use of better ultrasound technology and the subsequent increased detection of small nodules that have not been found in historical series [4]. We communicate a case of Leydig cell tumor with atypical evolution marked by maintaining of moderately elevated estradiol serum level four years after unilateral orchiectomy.

## Case presentation

**Figure 1:**
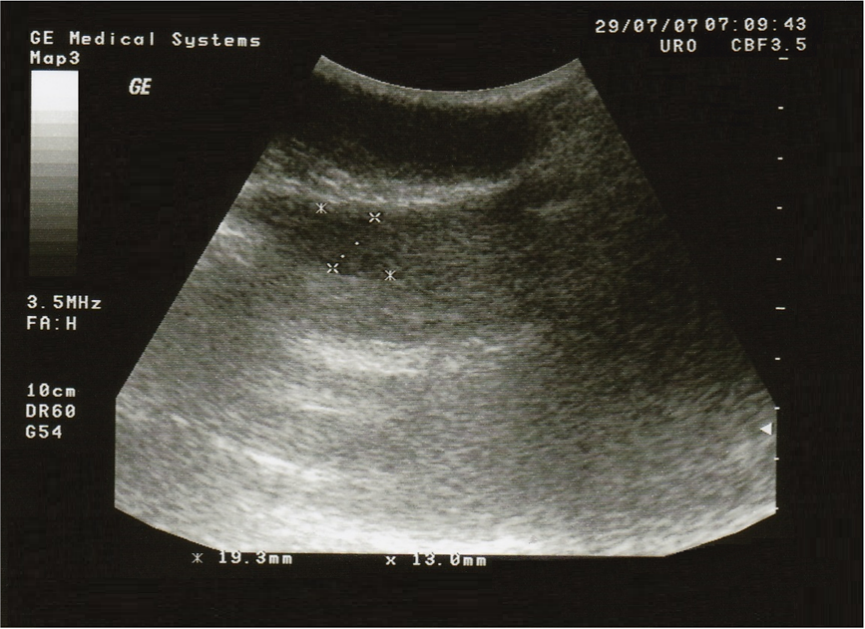
**Ultrasound of a right testis with Leydig cell tumor.** The tumor appears as a hypoechoic non-homogeneous mass within the testicular parenchyma.

**Figure 2:**
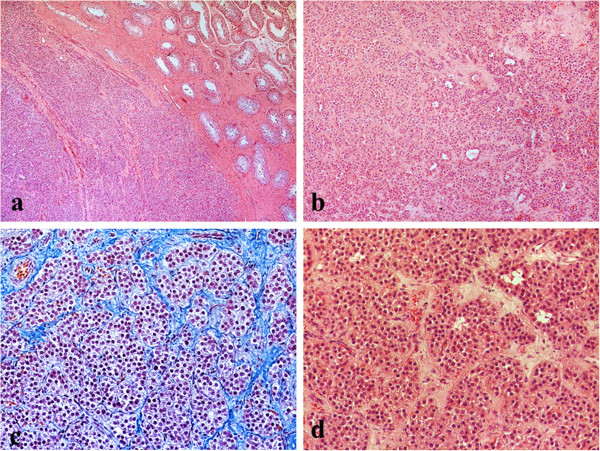
**Leydig cell tumors of the testis. a**. The tumor was surrounded by an incomplete, dense lamellar area. In non-neoplastic testicular tissue, the seminiferous tubules showed Sertoli cells with complete spermatogenesis, hematoxylin-eosin stain x10 **b**. No evidence of necrosis, or vascular invasion was seen. Stroma was fibrous with prominent vascularity, hematoxylin-eosin stain x10 **c**. The testicular tumor was composed of nests, insular or pseudotubular pattern, hematoxylin-eosin and methylene blue stain x40 **d**. The tumor cells were large, polygonal, with acidophilic to vacuolated cytoplasm and regular round nuclei, some with visible nucleoli. Mitoses were scarce, hematoxylin-eosin stain x40.

A 29-year-old Caucasian man presented to the endocrinology department with a recent right testicular mass observed at self-examination. The patient was born with right cryptorchidism. The right testis moved down into the scrotum before the age of two years old, without medical treatment. His family history revealed a lung cancer at his paternal grandfather. On physical examination the right testis was 6.5 × 3.5 cm in size, with a palpable tumoral mass of approximately 1.93 × 1.30 cm in size. No other sign, including gynecomastia or swelling of superficial lymph nodes was observed. Scrotal ultrasonography revealed a non-homogenous hypoechogenic tumoral mass in the right testis (Figure 1). Tumor markers such as alpha-fetoprotein (AFP) and β-human chorionic gonadotropin (β-hCG) were negative, and hormonal investigations were normal (Table 1). Surgery was indicated but the patient refused. He returned to the endocrinology department after two years with scrotal pain in the last month. One year before the patient has successfully fathered a child. Over these two years the patient overgrowth 30 kilos (at initial examination weight 90 kilos/height 195 cm). On physical examination the testicular tumor mass has not changed in size and scrotal ultrasonography had the same aspect, but gynecomastia was present. The tumor markers (AFP and β-hCG) were also negative and hormonal investigations were normal, except moderately elevated estradiol level (E2) and low normal level of testosterone (T) (Table 1). This time the patient accepted surgery. With a diagnosis of right testicular tumor, radical right high orchiectomy was performed and the specimen has been submitted for histopathological examination. The final diagnosis of benign Leydig cell tumor of testis was given (Figure 2). Immunohistochemical profile showed diffuse positive staining for Calretinin, Vimentin and CD99 and negative staining for Synaptophysin and Cytokeratin AE1/AE3. Hormonal investigations made after surgery showed still the persistence of a moderate increase of serum level of E2, while tumor markers AFP and β-hCG were still negative (Table 1). The postsurgical evolution was favorable. With diet and physical activity, the patient returned to normal weight one year after the unilateral orchiectomy. Without further treatment, the patient was evaluated yearly. The blood tests including tumor markers and hormonal investigations, along with scrotal ultrasound, abdominal and pelvis computed tomography (CT) scan were normal, except E2 serum level which was moderately increased each time (Table 1). The abdominal CT scan performed, four years after surgery, showed subdiaphragmatic micro lymphadenopathy (5–10 mm).

**Table 1:** **Tumor markers and hormonal evaluation at baseline, after surgery, and in the follow-up**

	2 y	Before	After	1 y	2 y	3 y	4 y	Normal range for age
	Eralier	Surgery	Surgery	After surgery	After surgery	After surgery	After surgery	
AFP, ng/ml	2.26	1.80	2.88	2.16	3.46	2.68	3.20	< 44
β-hCG, mIU/ml	0.10	0.06	0.09	0.08	0.20	0.16	0.13	0.5-2.67
Total testosterone, ng/ml	4.23	1.80	2.40	4.60	6.26	6.60	4.40	1.75-7.81
Estradiol, pg/ml	26.31	80.22	61.31	78.4	62.43	75.56	78.96	< 47
LH, mIU/ml	5.34	1.76	2.40	4.58	5.32	3.67	4.84	1.24-8.62
FSH, mIU/ml	4.23	3.80	2.49	2.80	4.12	2.74	3.85	1.27-19.26
17-KS, mg/24 hours	23.4	17.9	18.75	18.8	22.3	19.6	21.4	17-25
17-HOCS, mg/24 hours	8.4	10.22	9.07	8.8	7.40	9.22	8.94	2-10
Progesterone, ng/ml	-	-	0.09	-	-	-	-	0.10-0.84
TSH, μUI/ml	-	3.14	2.9	2.5	-	-	-	0.5-4.5

*Abbreviations:* *AFP* alpha-fetoprotein, *β-hCG* β-human chorionic gonadotropin, *FSH* follicle-stimulating hormone, *LH* luteinizing hormone, *17-KS* urinary 17-ketosteroids, *17-HOCS* urinary 17-hydroxycorticosteroid, *TSH* Thyroid-stimulating hormone.

## Discussion

The etiology of LCTs is unknown and it seems to be heterogeneous. Furthermore, the molecular basis of LCTs is poorly understood. Some studies showed a possible role of genetic factors in the etiology of this tumor. Interestingly, genetic mutations identified to date in child and adult were different, and in some cases have been associated with other cancers [5].

Some authors have reported in Leydig tumors in adult a somatic activating mutation in the guanine nucleotide-binding protein α gene, which may result in tumor development, leading to overexpression on the inhibin alpha subunit and hyperactivity of the testosterone biosynthetic pathway [6]. Also, in adult, it has been reported inherited fumarate hydratase mutation which appear to cause tumor growth through activation of the hypoxia pathway [7]. Alterations in local stimuli, including müllerian-duct inhibitory factor, inhibin, growth factors, and temperature, may also create favorable conditions for tumorigenesis [8].

LCTs are usually unilateral. However, 3-10% of them have been estimated to be bilateral and 15% may extend beyond the testis at the time of presentation. Origin from testicular Leydig cells is demonstrable in most cases, but rarely they may originate from the epididymis [9]. Although these tumors arise at any age, they affects mostly men between 20–60 years old.

In our case the tumor was unilateral in right testis and the patient belongs to the age group with the highest incidence.

LCTs may be an incidental finding of a testicular mass on scrotal ultrasonography performed for other conditions [10]. Except for the testicular tumor mass present in all cases, other signs and symptoms may be present in different degrees (pain in the testis, enlargement of a testicle, heaviness in the scrotum, and gynecomastia). Azoospermia and infertility are uncommon and, if exist they are reversible after removal of the tumor [11].

Ultrasound of scrotum is very useful to confirm the diagnosis of testicular tumor [12], but it cannot differentiate between a benign and a malignant tumor [13]. In our case scrotal ultrasonography has revealed a non-homogenous hypoechogenic tumoral mass in the right testis.

In patients with LCTs the blood tests for tumor markers (AFP, β- hCG, and lactate dehydrogenase) are negative.

The most common hormones secreted by LCTs are testosterone and estrogens. In most cases, adults have non functional testicular masses.

In our case tumor markers were always negative. Hormonal profile was initially normal. Two years after the discovery of the tumor the occurrence of gynecomastia and moderately elevated E2 serum level associated with decreased T to lower normal serum value was observed. After unilateral orchiectomy the blood tests were normal, except E2 serum level which was moderately increased each time (Table 1).

The use of fine needle aspiration for diagnosis may be an alternative to more invasive biopsy procedures in the preoperative diagnosis of this rare testicular tumor [14].

CT scan of the abdomen and pelvis and chest radiography are indicated especially if malignancy is suspected. In our case, except the tumor in the right testis, CT scan was normal.

Orchiectomy is the initial treatment for LCTs, with or without removal of nearby lymph nodes (lymphadenectomy). Today, some authors suggest a more conservative therapy. Testis sparing surgery has proved to be a feasible and safe choice and could be regarded as first-line therapy in cases of benign, small tumors (under 25 mm), and young men [15–19].

Although orchiectomy is curative in approximately 90% of cases, the remaining patients can develop metastases refractory to chemotherapy and radiations. Radical orchiectomy remains in use if malignancy is suspected [20].

In our case, based on family and personal medical history, radical right high orchiectomy was performed.

The pathological exam and immunohistochemistry are essential for the diagnosis and for the next steps in treatment. In our case, no histopathological feature suggestive of malignancy was seen and the final diagnosis of benign Leydig cell tumor of testis was given. The results of immunoreactive tests were consistent with the existing literature (Table 2) [21–28].

**Table 2:** **Immunohistochemical staining results of Leydig cell tumors**

	Marker	Immunoreactivity
**Benign Leydig cell tumors**	Inhibin A	+
	Calretinin*	+
	Melan A (Mart-1)	+
	Vimentin*	+
	S-100	+
	Synaptophysin*	+/−
	Chromogranin	+
	CD99*	+
	P- /N-Cadherin	+
	RLF/INSL3	+
	StAR protein	+
	SF-1	+
	Cytokeratin*	-
	PLAP	-
	CD-30	-
	Oct3/4	-
**Malignant Leydig cell tumors**	Ki67	+
	p53	+
	Bcl2	+

*Abbreviations:* *RLF/INSL3* Relaxin-like factor/Insulin-like 3, *StAR-protein* Steroidogenic Acute Regulatory protein, *SF-1* Steroidogenic factor 1, *PLAP* Placental Alkaline Phosphatase. *Immunohistochemical tests performed in our case.

While benign, LCTs have malignant potential in about 10% of cases with metastatic forms, particular to the lymph nodes, especially the retroperitoneal and inguinal nodes (70%), liver (45%), lungs (40%), and bone (25%) [29]. The metastatic type occurs exclusively in adults and is more common in older patients with an average age of more than 40 years. The risk of malignancy in the undescended testis is 4 to 10 times higher than that in the general population but the most common type of testicular cancer occurring in undescended testes is seminoma [30].

Palazzo *et al.* have suggested that the majority of LCTs are diploid and the less common malignant tumors are typically aneuploid, and that deoxyribonucleic acid flow cytometric findings can be useful as a prognostic indicator in these tumors [31].

In our case all clinical, hormonal, immunocytochemical, and pathological findings have supported the diagnosis of benign LCT.

Initially, starting from negative tumor markers and normal hormonal profile, tumoral mass seemed to be a non functional Leydig cell tumor, the only detectable element has been testicular tumor mass. After two years the occurrence of gynecomastia and moderately elevated E2 serum level associated with decreased testosterone to lower normal serum value was observed which led to a new considerations of the case presented. Leydig cells can synthesize both androgens and estrogen, and this cells contain the aromatase enzyme necessary to convert irreversibly testosterone to estradiol [32]. There have been reported cases of Leydig cell tumor which demonstrated only an increase of serum E2 and suppression of serum testosterone as a consequence of increased aromatase activity in tumoral Leydig cells [33]. The direct inhibitory action of elevated E2 on the enzymes involved in steroidogenesis and the negative feedback action on luteinizing hormone secretion of the E2 possibly caused the suppression of serum testosterone [34]. Due to the significant increase in weight of the patient, in our case hormonal changes were rather considered a consequence of aromatization in adipose tissue of testosterone to estradiol and gynecomastia as a consequence of increased levels of estradiol and change of estrogen/androgen ratio. This assumption was confirmed by maintaining high levels of estradiol after unilateral orchiectomy, because it was demonstrated that after removing the tumor, in most cases, hormone levels return to normal, which in the case presented did not. Therefore has been indicated to the patient the weight loss in the hope that he will return to normal levels of estradiol and gynecomastia will disappear. Surprisingly, after the return to normal weight, estradiol levels continued to be slightly increased in the absence of any identifiable factors that could explain the maintenance of high levels of estradiol, such as red meat abuse consumption [35].

Statistics show that LCTs are rarely malignant and they correlate with increasing age of the patient, tumor size (over 5 cm) and increased tumor mitotic index. These data were missing in our case. In 2002 Maeda *et al*. raported on an adult patient with a larger testicular tumor in whom metastasis of Leydig cell tumor was suggested by elevated serum estradiol 9 years after the removal of the primary tumor [36].

We cannot yet draw a conclusion. For four years our patient remained free of disease, with no evidence of recurrence or metastasis, but persistent moderate high levels of estradiol and the result of the last abdominal CT scan (subdiaphragmatic micro lymphadenopathy) do not definitely exclude this possibility. It is known that changes in hormonal assay can be used as a marker of tumor recurrence in patients followed. Furthermore, the patient has a family history of cancer and at birth he had cryptorchidism. These data require careful monitoring and correct therapeutic decisions. Although estradiol levels are not suggestive for a malignant lesion, imaging investigations are of great interest, and need to be repeated periodically. Moreover, endocrine evaluation is useful for patients with a Leydig cell tumor in whom endocrinological abnormality is demonstrated. Many times was told that the next step in such cases is “wait and see”, which for our patient seems to be the most appropriate thing.

## Conclusions

Leydig cell tumors are uncommon neoplasms arising from gonadal stroma. It is critical for physicians to remember and do not overlook the possibility of this rare tumor. The self-examination of testicles appears to be a very important step for the diagnosis of testicular tumors.

In Leydig cell tumors orchiectomy is the elected therapeutic decision. In the absence of any sign of malignancy, long-term follow-up is necessary to exclude recurrence or metastasis. Because we cannot always identify the reasons of persistent elevated E2 serum level after unilateral orchiectomy for Leydig cell tumors, the endocrine profile and imaging investigations need to be repeated periodically.

## Consent

Written informed consent was obtained from the patient for publication of this Case Report and any accompanying images. A copy of the written consent is available for review by the Editor-in-Chief of this journal.

## Abbreviations

LCTs: Leydig cell tumors
AFP: alpha-fetoprotein
β-hCG: β-human chorionic gonadotropin
E2: Estradiol
T: Testosterone
CT: Computed tomography.
